# Supplementary material for: Knowledge and Competence Regarding the Management of Chronic Kidney Disease among Family Medicine Professionals in the Eastern Province of Saudi Arabia: A Cross-Sectional Study
Source: Int J Environ Res Public Health. 2024 Jul 6;21(7):880. doi: 10.3390/ijerph21070880 (PMC11276965; doi:10.3390/ijerph21070880)
Supplement: Supplementary file 1 [file ijerph-21-00880-s001.zip › ijerph-3024501-Supplementary Material.pdf]

International Journal of
Environmental Research and Public Health

MDPI

Supplementary material:

Table S1. Assessment of knowledge and confidence regarding the management of chronic kidney disease among family medicine professionals (N=71).

Statement	n (%)
1. Understanding the significance and importance of urine albumin-creatinine ratio (uACR) testing in individuals living with diabetes	
• Not confident about this subject	01 (01.4%)
• Some degree of confidence would like to know more	04 (05.6%)
• Confident to practice in this area with support	09 (12.7%)
• Confident to practice in this area without support	17 (23.9%)
• Fully confident in this area and could teach others	40 (56.3%)
2. Ability to interpret Urine Albumin-to-Creatinine Ratio	
• Not confident about this subject	01 (01.4%)
• Some degree of confidence would like to know more	04 (05.6%)
• Confident to practice in this area with support	09 (12.7%)
• Confident to practice in this area without support	24 (33.8%)
• Fully confident in this area and could teach others	33 (46.5%)
3. Knowledge of stages of kidney disease according to estimated GFR	
• Not confident about this subject	01 (01.4%)
• Some degree of confidence would like to know more	04 (05.6%)
• Confident to practice in this area with support	05 (07.0%)
• Confident to practice in this area without support	19 (26.8%)
• Fully confident in this area and could teach others	42 (59.2%)
4. Knowledge of the criteria for diagnosis of chronic kidney disease (CKD) and diabetic kidney disease (DKD)	
• Not confident about this subject	01 (01.4%)
• Some degree of confidence would like to know more	04 (05.6%)
• Confident to practice in this area with support	10 (14.1%)
• Confident to practice in this area without support	22 (31.0%)
• Fully confident in this area and could teach others	34 (47.9%)
5. Knowledge of appropriate next steps in treatment after diagnosis	
• Not confident about this subject	01 (01.4%)
• Some degree of confidence would like to know more	05 (07.0%)
• Confident to practice in this area with support	16 (22.5%)
• Confident to practice in this area without support	22 (31.0%)
• Fully confident in this area and could teach others	27 (38.0%)
6. Understanding of how to predict CKD prognosis using albuminuria and estimated GFR categories (using KDIGO guidelines)	
• Not confident about this subject	03 (04.2%)
• Some degree of confidence would like to know more	13 (18.3%)
• Confident to practice in this area with support	10 (14.1%)

• Confident to practice in this area without support	26 (36.6%)
• Fully confident in this area and could teach others	19 (26.8%)
7. Recognising the possible signs and symptoms of more advanced CKD	
• Not confident about this subject	01 (01.4%)
• Some degree of confidence would like to know more	05 (07.0%)
• Confident to practice in this area with support	15 (21.1%)
• Confident to practice in this area without support	24 (33.8%)
• Fully confident in this area and could teach others	26 (36.6%)
8. Awareness of kidney disease as a risk multiplier, increasing the risk of cardiovascular disease (CVD) and other complications, and the interconnectivity of the renal system with CVD and diabetes	
• Not confident about this subject	01 (01.4%)
• Some degree of confidence would like to know more	05 (07.0%)
• Confident to practice in this area with support	10 (14.1%)
• Confident to practice in this area without support	22 (31.0%)
• Fully confident in this area and could teach others	33 (46.5%)
9. Able to select appropriate management (treatments and offer lifestyle advice) for preventing or slowing the progression of CKD	
• Not confident about this subject	02 (02.8%)
• Some degree of confidence would like to know more	04 (05.6%)
• Confident to practice in this area with support	13 (18.3%)
• Confident to practice in this area without support	30 (42.3%)
• Fully confident in this area and could teach others	22 (31.0%)
10. Understanding blood pressure targets	
• Not confident about this subject	01 (01.4%)
• Some degree of confidence would like to know more	03 (04.2%)
• Confident to practice in this area with support	04 (05.6%)
• Confident to practice in this area without support	22 (31.0%)
• Fully confident in this area and could teach others	41 (57.7%)
11. Understand the use of treatments such as angiotensin-converting enzyme inhibitors or angiotensin II receptor blockers and their renal benefits	
• Not confident about this subject	01 (01.4%)
• Some degree of confidence would like to know more	01 (01.4%)
• Confident to practice in this area with support	07 (09.9%)
• Confident to practice in this area without support	23 (32.4%)
• Fully confident in this area and could teach others	39 (54.9%)
12. Confidently initiating diabetes medications that have particular benefits in DKD such as SGLT2-Is and glucagon-like peptide 1 receptor agonists	
• Not confident about this subject	04 (05.6%)
• Some degree of confidence would like to know more	04 (05.6%)
• Confident to practice in this area with support	08 (11.3%)
• Confident to practice in this area without support	19 (26.8%)

• Fully confident in this area and could teach others	36 (50.7%)
**Total confidence score (mean ± SD)**	**49.5 ± 9.66**
Level of confidence	
• Low	03 (04.2%)
• Average	17 (23.9%)
• High	51 (71.8%)
